# Newly Emerged Serotype 1c of *Shigella flexneri*: Multiple Origins and Changing Drug Resistance Landscape

**DOI:** 10.3390/genes11091042

**Published:** 2020-09-03

**Authors:** Pawan Parajuli, Bui Quang Minh, Naresh K. Verma

**Affiliations:** 1 Division of Biomedical Science and Biochemistry, Research School of Biology, The Australian National University, Canberra, ACT 2601, Australia; pawan.parajuli@anu.edu.au; 2 Research School of Computer Science & Research School of Biology, The Australian National University, Canberra, ACT 2601, Australia; m.bui@anu.edu.au

**Keywords:** *Shigella flexneri* serotype 1c, bacteriophage, serotype evolution, multidrug resistance

## Abstract

Bacillary dysentery caused by *Shigella flexneri* is a major cause of under-five mortality in developing countries, where a novel *S. flexneri* serotype 1c has become very common since the 1980s. However, the origin and diversification of serotype 1c remain poorly understood. To understand the evolution of serotype 1c and their antimicrobial resistance, we sequenced and analyzed the whole-genome of 85 clinical isolates from the United Kingdom, Egypt, Bangladesh, Vietnam, and Japan belonging to serotype 1c and related serotypes of 1a, 1b and Y/Yv. We identified up to three distinct O-antigen modifying genes in *S. flexneri* 1c strains, which were acquired from three different bacteriophages. Our analysis shows that *S. flexneri* 1c strains have originated from serotype 1a and serotype 1b strains after the acquisition of bacteriophage-encoding *gtrIc* operon. The maximum-likelihood phylogenetic analysis using core genes suggests two distinct *S. flexneri* 1c lineages, one specific to Bangladesh, which originated from ancestral serotype 1a strains and the other from the United Kingdom, Egypt, and Vietnam originated from ancestral serotype 1b strains. We also identified 63 isolates containing multiple drug-resistant genes in them conferring resistance against streptomycin, sulfonamide, quinolone, trimethoprim, tetracycline, chloramphenicol, and beta-lactamase. Furthermore, antibiotic susceptibility assays showed 83 (97.6%) isolates as either complete or intermediate resistance to the WHO-recommended first- and second-line drugs. This changing drug resistance pattern demonstrates the urgent need for drug resistance surveillance and renewed treatment guidelines.

## 1. Introduction

Shigellosis or bacillary dysentery is caused by members of the *Shigella* genus, which are human-specific enteric pathogens. *Shigella* species are closely related to *Escherichia coli* but have specific virulence traits [1]. Historically, shigellosis was associated with significant morbidity and mortality during all major conflicts of the 19th and 20th centuries, during natural disasters, times of political upheaval, and subsequent refugee crises [2,3,4]. Shigellosis continues to be a major etiological agent of food and water-borne illness in many developing countries due to poor sanitation facilities and a lack of safe drinking water [5]. A recent study estimated that there are at least 190 million annual shigellosis cases and 70,000 deaths worldwide [6]. The majority of these deaths occurred in Sub-Saharan Africa and South Asia and involved children less than five years of age [7,8,9]. Recently, *Shigella* transmission dynamics have expanded to developed countries. This is largely due to increased international travel and sexual practices among men who have sex with men (MSM) [10].

**Figure 1:**
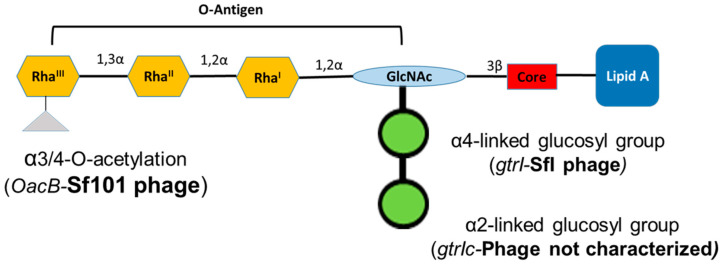
Schematic representation of the lipopolysaccharide of *Shigella flexneri* serotype 1c. Each tetrasaccharide unit of O-antigen has one *N*-acetylglucosamine (GlcNAc) and three rhamnose sugars (Rha). All serotype 1c strains have two glucosyl groups attached to GlcNAc and some of them have an additional acetyl group attached to Rha^III^.

*Shigella flexneri* is the predominant species of *Shigella* causing shigellosis in developing countries in Asia [9], Latin America [11,12] and Africa [13]. The *S. flexneri* serotypes, except for serotype 6, consist of a polysaccharide backbone with the repeating tetrasaccharide units of three rhamnoses and an *N*-acetylglucosamine (GlcNAc) residue in the O-antigen component of the bacterial lipopolysaccharide (LPS) [14]. The modifications to the basic O-antigen backbone by the addition of glucosyl, O-acetyl, or phosphoethanolamine groups to one or more sugars give rise to at least 19 serotypes of *S. flexneri* [15] (Figure 1).

*S. flexneri* serotype 1c is an emerging serotype in developing countries [16]. The serotype 1c was first identified during the 1980s and since then, it has become the most common *S. flexneri* serotype in multiple geographic regions [17,18,19,20]. This serotype has a unique O-antigen structure, comprised of a disaccharide (two-glucosyl group)-α-D-Glc*p*-(1➔2)-α-D-Glc*p* linked to O4 of the GlcNAc residue [21]. The glucosylation in *S. flexneri* is performed by a bacteriophage-acquired operon containing three genes; *gtrA*, *gtrB* and *gtr(type)*. The products of first two genes, *gtrA* and *gtrB,* are highly conserved among different *S. flexneri* serotypes and are involved in transferring the glucosyl group from the cytoplasm to the periplasm [22]. The *gtr(type)* gene (where ‘type’ refers to *S. flexneri* serotype) is unique to each serotype and encodes a serotype-specific glucosyltransferase that attaches the glucosyl group to a specific sugar in the tetrasaccharide repeat unit [23]. The addition of two glucosyl groups is due to the presence of two separate *gtr* operons in the serotype 1c chromosome. The addition of the first glucosyl group is mediated by the *gtrI* operon carried by the SfI bacteriophage, which is also the basis of O-antigen modification in *S. flexneri* serotypes 1a and 1b. However, the second glucosyl group is mediated by the *gtrIc* operon, carried by a different, uncharacterized and distantly related bacteriophage [24]. Recently, the complete genome analysis of *S. flexneri* serotype 1c strain Y394 showed that these two phage regions are arranged as a cryptic prophage at 2 Mb apart [16]. Furthermore, some of the serotype 1c strains also have additional O-antigen modification, due to the addition of the O-acetyl group mediated by *oacB* gene, encoded by the bacteriophage Sf101 [25].

Although the serotype 1c is one of the most common *S. flexneri* serotypes in developing countries, the diversity and evolution of serotype 1c remain poorly understood. Until recently, *S. flexneri* phylogenetic studies only used a handful of housekeeping genes [26,27]. Recently, sequencing technologies have provided an unprecedented amount and quality of genome sequence data at a reasonable cost. The high-resolution whole-genome sequences have enabled us to capture a detailed population structure and complete genomic variation within species. In this study, we performed whole-genome sequencing of 85 different clinical isolates: 66 serotype 1c, 8 serotype 1a, 7 serotype 1b and 4 serotype Y/Yv strains from the United Kingdom (UK), Egypt, Bangladesh, Vietnam, and Japan. We set out to understand the molecular basis of serotype 1c evolution, alongside the phylogenetic and antibiotic resistance gene analysis. This analysis allows us to understand the clonal relationships of different serotype 1c and related strains. It also provides valuable information on the drug resistance for shigellosis treatment.

## 2. Materials and Methods

### 2.1. Bacterial Strains

The *S. flexneri* strains and their sources included in this study are listed in Supplementary Table S1. After receiving the strains from the host laboratory, they were cultured overnight on Luria Bertani (LB) agar plates at 30 °C to maintain the virulence plasmid. The *S. flexneri* serotypes were confirmed by slide agglutination using commercially available group and type antisera (Denka Seiken, Tokyo, Japan) and the serotype-1c specific monoclonal antibody MASF IC (Reagensia AB, Stockholm, Sweden). The bacterial strains were then stored in glycerol stocks and maintained at −80 °C.

### 2.2. DNA Preparation and Sequencing

The bacterial strains were streaked out from glycerol stocks using a sterile loop onto LBA plates and incubated overnight at 37 °C. An isolated single colony was selected from the LBA plates and inoculated in LB broth and grown aerobically (180 rpm) at 37 °C. 1 mL of overnight cultures was subsequently used for bacterial DNA extraction using the Illustra^TM^ Bacteria Genomic Prep Mini Spin Kit (GE Healthcare, Buckinghamshire, UK) according to the manufacturer’s instructions. The libraries were constructed using the Nextera XT DNA library preparation kit (Illumina, Inc., San Diego, CA, USA) and sequenced on Illumina MiSeq instrument using MiSeq v3 2 × 300 bp paired-end protocol. Both the sequencing and library preparation were performed at either the Ramaciotti Centre for Genomics (University of New South Wales) or the Biomolecular Resources Facility, John Curtin School of Medical Research (Australian National University).

### 2.3. Bioinformatics Analysis

The quality of MiSeq reads was assessed using FastQC v0.11.5 [28] and trimmed accordingly using Trimmomatic v0.36 [29]. The analysis of bacteriophage region carrying *gtrI* and *gtrIc* operons was performed using the reference genome of *S. flexneri* serotype 1a strain 0439 (GenBank Accession: CP020342) and *S. flexneri* serotype 1c strain Y394 (GenBank Accession: CP020753), respectively. The quality trimmed raw reads were mapped to the above reference using Burrows-Wheeler Aligner (BWA) [30], SAMtools [31] and visualized using IGV [32].

For Pan-genome and phylogenetic analysis, the whole-genome data for each of the isolates were assembled de novo using Velvet assembler [33]. Each of these genomes including the genomes obtained from the publicly available repositories was annotated using Rapid Prokaryotic Genome Annotation (Prokka) [34] to reduce the effects of biases. PHASTER (PHAge Search Tool - Enhanced Release) database [35] was used to find the bacteriophage-encoded regions within each genome. Following the Prokka annotation, the genes that were common in all compared strains (core genes) and accessory genes were extracted using the Roary pipeline [36]. The core gene alignment was used to reconstruct a phylogenetic tree using IQ-TREE [37] based on the best-fit model determined by ModelFinder [38]. The branch supports were assessed with the ultrafast bootstrap with 1000 replicates [39] and the Shimodaira-Hasegawa-like approximate likelihood ratio test (SH-aLRT) [40]. The image files were generated using SnapGene Viewer (Version 3.3.4, GSL Biotech LLC, San Diego, USA), Adobe Illustrator (Version 21.1.0, Adobe Systems, San Jose, CA, USA), Artemis Comparison Tool [41] and FigTree v1.4.4 [42].

### 2.4. Antimicrobial-Resistant Genes and Susceptibility Testing

The antimicrobial genes present in each bacterial strains were detected using the ResFinder database [43]. The antibiotic susceptibility pattern of each strains was determined using the disk diffusion method (Kirby-Bauer) [44]. We tested drug resistance against a range of modern antibiotics (Oxoid, UK) including ampicillin (10 µg), azithromycin (15 µg), ceftriaxone (30 µg), chloramphenicol (30 µg), ciprofloxacin (5 µg), kanamycin (30 µg), nalidixic acid (30 µg), streptomycin (25 µg), tetracycline (30 µg) and trimethoprim/sulfamethoxazole (1.25/23.75 µg).

## 3. Results

### 3.1. Integration of O-Antigen Modifying Bacteriophages in Serotype 1c Strains

**Figure 2:**
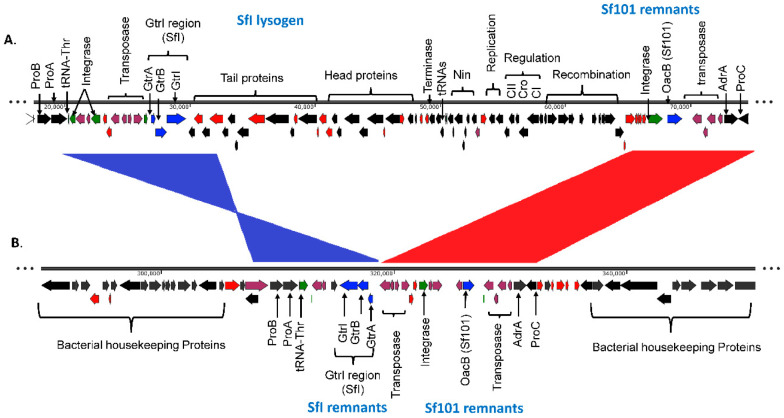
Comparison of SfI phage region in *S. flexneri* serotype 1a and *S. flexneri* serotype 1c. The two genomes are shown to scale. The numbers below the scale bar are the positions in the genome in base pairs. The direction of arrows indicates the orientation of open reading frames (ORFs). The labels show the major proteins encoded by the ORFs. The color code denotes the ORF types: red, hypothetical protein; plum, transposase and IS elements; green, tRNA and integrase; black, annotated proteins and blue, *gtr,* and *oacB* genes. (**A**). Complete SfI lysogen sequence in *S. flexneri* serotype 1a strain 0439. The SfI prophage is integrated within the *tRNA-Thr* gene. The prophage contains *gtrI* operon as well as all the essential genes required for lytic cycle. The remnants of Sf101 phage which contains *oacB* gene is present adjacent to the SfI prophage. (**B**). Remnants of SfI phage in *S. flexneri* 1c strain Y394. Y394 possesses a few genes of SfI prophage including *gtrI* operon but the orientation is in inverted synteny which is shown in blue. The Sf101 phage remnants are also present in Y394 adjacent to SfI remnants with collinear synteny which is shown in red.

**Figure 3:**
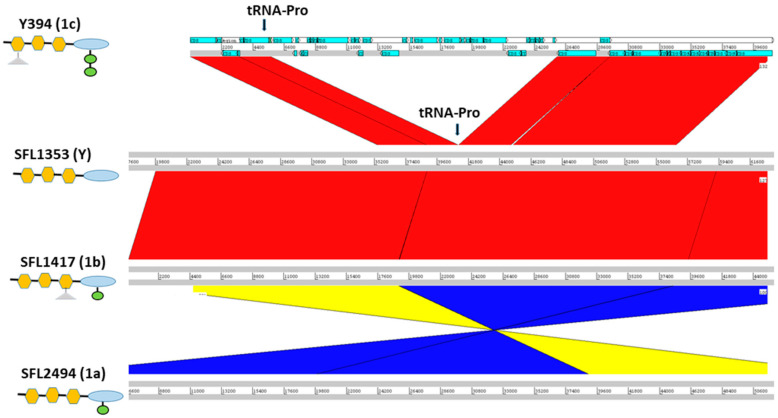
Cryptic SfIC phage region in *S. flexneri* serotypes 1c, Y, 1b and 1a from panel top to bottom, respectively. The left panel shows the strain names with the respective serotype in the parenthesis. The schematic representation of the O-antigen structure in each serotype is shown below the strain name with symbols as of Figure 1. The genomic regions are depicted as horizontal gray lines interspersed with regions of collinear (red) and inverted (yellow/blue) synteny. The blue blocks in Y394 denote the coding sequences (CDS) present. The cryptic SfIC phage region integrated within *tRNA-Pro* gene in Y394 is missing in serotype Y, serotype 1b and serotype 1a. However, other genes in the upstream and downstream regions of the *tRNA-Pro* gene are intact in serotype Y, 1b, and 1a.

The first glucosylation in all the *S. flexneri* serotype 1c strains is mediated by the *gtrI* operon, which is carried by a previously characterized bacteriophage SfI [45]. The analysis of SfI phage remnants including the presence of *gtrI* operon was investigated using *S. flexneri* serotype 1a strain 0439 (GenBank Accession: CP020342.1) which has a complete copy of SfI prophage in its genome. Our analysis showed that the SfI phage specifically integrated into the *tRNA-Thr* gene at *proA* locus in all the 1a, 1b and 1c serotypes. The majority of SfI phage genes were deleted while leaving only 12 % (5,612 bp of 38,389 bp) of SfI phage sequence including 2,803 bp of the *gtrI* operon in all the 1b (*n* = 7), 1c (*n* = 66) and the majority of 1a (7 out of 8) strains (Supplementary Figure S1, Supplementary Material online). However, one of the serotype 1a strains (SFL2494, UK) contained a complete SfI phage sequence. Interestingly, 8 out of 66 1c strains and all 1a and 1b strains also harbored the *oacB* gene adjacent to the SfI phage region. The *oacB* gene is originally carried by a previously characterized bacteriophage Sf101 integrated into the *sbcB* gene locus and is responsible for O-acetylation in *S. flexneri* strains [25]. Two serotype 1c strains, SFL1683 and SFL1684 from Egypt, contained a complete copy of Sf101 bacteriophage. Those strains that have *oacB* gene adjacent to SfI phage also possessed the partial Sf101 integrase sequence in the *sbcB* locus. This suggests that the *oacB* gene has been translocated to the SfI phage region from its original integration site by means of IS elements/transposases flanking the *oacB* gene (Figure 2). To investigate the presence of complete phage carrying *gtrIc* operon (referred to as SfIC), the individual PHASTER search [35] was performed using the de novo assembled genome sequences of the individual *S. flexneri* serotype 1c strains. This analysis showed an absence of complete SfIC prophage. The sequence reads of all the strains were mapped using the reference genome of *S. flexneri* serotype 1c (Y394), which consists of 20kb SfIC cryptic phage sequences including *gtrIc* operon. All of the serotype 1c strains had identical SfIC phage remnants integrated into the *tRNA-Pro* gene (Supplementary Figure S2). However, there were no SfIC remnant sequences present in serotype 1a, 1b and Y/Yv strains (Figure 3).

### 3.2. Pangenome and Phylogenetic Analysis

We performed the pangenome and phylogenetic analysis using a subset of 75 samples (out of 85) with high coverage (>85%) with the Y394 reference genome. We additionally included 10 published *S. flexneri* complete genomes in the analysis, totaling 85 samples. The analysis identified a large *S. flexneri* pangenome with 7,991 homologous groups of genes, with 3244 (40.6%) core genes, which were present in all the compared isolates (Supplementary Table S2). The presence of a large pangenome with less than half of core genes indicates the rapid evolution of this pathogen over a short time (Supplementary Figure S3).

**Figure 4:**
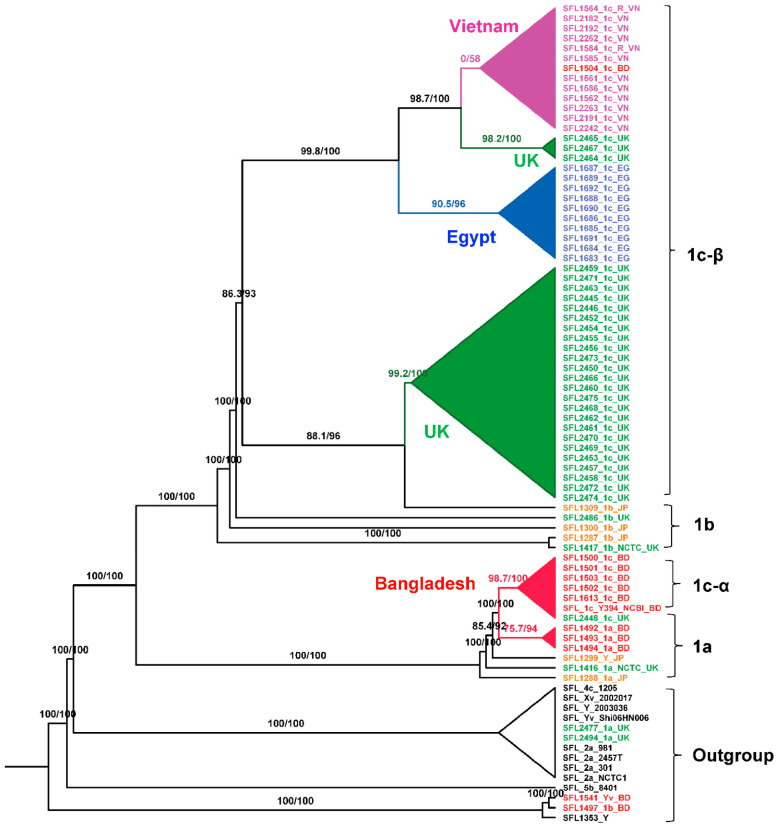
Maximum-Likelihood (ML) phylogeny for *S. flexneri* isolates. The phylogeny was based on the alignment of concatenated core genes with the recombination region removed. The ML tree was reconstructed using the general time-reversible model with a proportion of invariable sites (GTR + F + I) as the best-fit model. The analysis showed two distinct 1c lineages: 1c-α is specific to Bangladeshi isolates that was likely evolved from 1a strains; 1c-β is specific to the isolates from the UK, Egypt, and Vietnam. The labels indicate the strain number followed by the serotype and the country of isolation. The isolates from different sources were color-coded: red (Bangladesh), blue (Egypt), orange (Japan), green (UK), purple (Vietnam), and black (GenBank). The branch annotations are the Shimodaira-Hasegawa approximate likelihood ratio supports and the bootstrap supports (1000 pseudoreplicates). The branch lengths are not drawn to scale.

We carried out the phylogenetic analysis on the alignment of concatenated core genes using IQ-TREE version 2.0-rc1 [37] after removing recombinant regions using Gubbins [46] and manual removal of three rogue taxa (SFL1496, SFL1538, and SFL1315) with unstable positions in the tree. The branch supports were assessed with the Shimodaira-Hasegawa approximate likelihood ratio test (SH-aLRT) [40] and the ultrafast bootstrap (UFBoot) with 1000 pseudo-replicates [39]. The maximum-likelihood phylogeny (Figure 4) showed a clear clustering of the sequences based on the sampling countries with high branch supports for the tree backbone (SH-aLRT ≥ 80% and UFBoot ≥ 95%). Interestingly, Figure 4 revealed two independently evolved 1c lineages, which we denote as 1c-α and 1c-β. The 1c-α is specific to Bangladesh and was derived from the ancestral 1a strains after the acquisition of SfIC phage whereas the 1c-β clade was likely originated from the ancestral 1b strains after the acquisition of SfIC phage and is present in isolates from the UK, Egypt, and Vietnam. All the other *S. flexneri* serotypes included in the analysis formed an outgroup outside the serotype 1 lineage as expected (Figure 4 and Supplementary Figure S4).

### 3.3. Antibiotic Resistance

**Figure 5:**
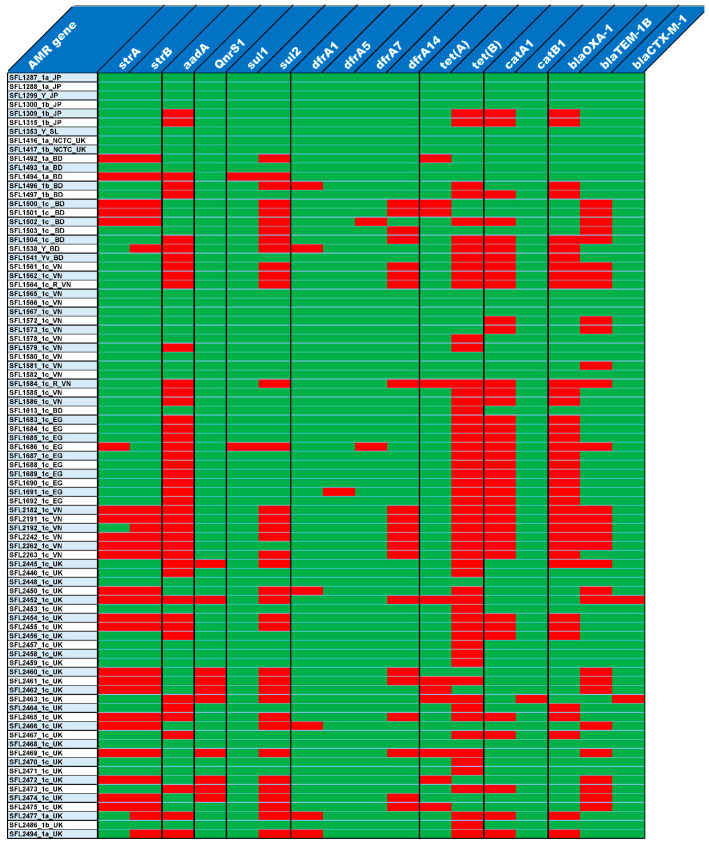
Antibiotic resistance gene profiles. The top row shows the list of antibiotic resistance genes and the left column shows the list of strains. The presence and absence of antibiotic resistance genes in each of the strains is shown in red and green colors, respectively.

**Figure 6:**
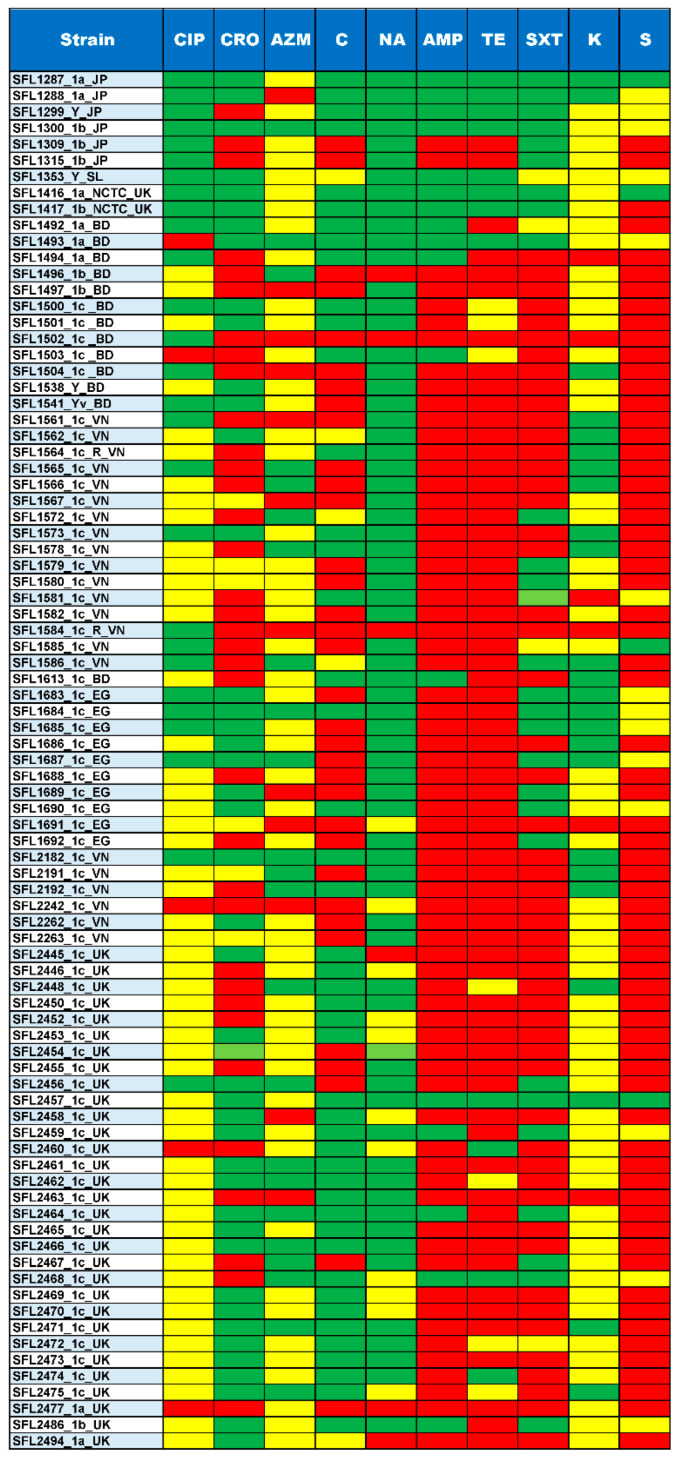
Summary of Antimicrobial susceptibility assay. The top row shows the antibiotics tested for each of the strains shown in the left column. The colors green, yellow and red indicate sensitive, intermediate and resistance against the antibiotics tested, respectively. The ten antibiotics tested include CIP, Ciprofloxacin; CRO, Ceftriaxone; AZM, Azithromycin; C, Chloramphenicol; NA, Nalidixic acid; AMP, Ampicillin; TE, Tetracycline; SXT, trimethoprim/sulfamethoxazole; K, Kanamycin and S, Streptomycin.

The majority of the 85 tested strains (73%) possessed multiple drug-resistant genes (*strA*, *strB*, *aadA*, *qnrS1*, *sul1*, *sul2*, *dfrA1*, *dfrA5*, *dfrA7*, *dfrA14*, *tet(A)*, *tet(B)*, *catA1*, *catB1*, *blaOXA-1*, *blaTEM-1B* and *blaCTX-M-1*) in their genomes (Figure 5). The genotypes were consistent with the phenotypes tested with the relevant antimicrobial compounds (Figure 6). Additionally, the antibiotic susceptibility patterns of drugs used for shigellosis treatment recommended by the World Health Organization [47] were examined. Unfortunately, most isolates (68% of samples) showed either resistant or intermediate susceptibility to first-line drugs (ciprofloxacin; CIP). Similarly, only half of the strains were susceptible to second-line drugs such as ceftriaxone (CRO) and one-third were susceptible to azithromycin (AZM). Surprisingly, one of the drugs-nalidixic acid (NA), which was discontinued due to increasing resistance, performed better than the currently recommended drugs with only 20% of isolates exhibiting resistance or intermediate susceptibility (Figure 6). This warrants continuous surveillance of antibiotic resistance in *Shigella* species.

## 4. Discussion

**Figure 7:**
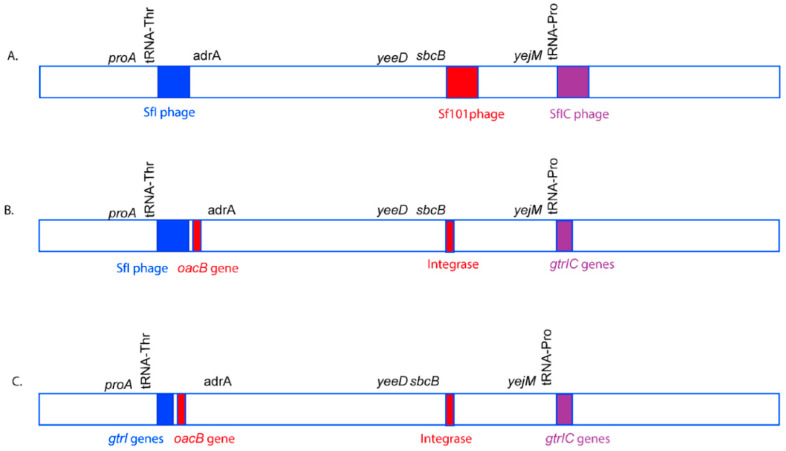
A model of O-antigen modifying phage acquisition in *S. flexneri* serotype 1c. The genomic region is not drawn to scale. The O-antigen modifying phages, SfI (blue), Sf101 (Red), and SfIC (Purple) integrate in *tRNA-Thr*, *sbcB* and *tRNA-Pro* genes, respectively in the *S. flexneri* genome to give rise to *S. flexneri* serotype 1c (**A**). However, over time, most of the structural genes of the phages Sf101 and SfIC are lost due to recombination events. The remnants of Sf101 and SfIC have only a few genes including genes required for serotype conversion. Moreover, the *oacB* gene has been translocated adjacent to SfI region with the help of transposases (**B**). Furthermore, over time, SfI phage also loses most of its structural components, remaining only a handful of genes including the O-antigen modifying genes. Hence, all the three phages SfI, Sf101 and SfIC remain as remnants in most of the current *S. flexneri* serotype 1c isolates (**C**).

The *S. flexneri* strains are divided into various serotypes based on O-antigenic variations in LPS caused by horizontally acquired genes from bacteriophages (*gtr* and *oac/oac*B) and/or plasmids (*opt)*. *S. flexneri* serotype 1c is unique to its origin as it had to undergo subsequent acquisition of *gtr* genes in background serotype 1a/1b strains in order to have two glucosyl group at the GlcNAc. The integration of these two phages was within the tRNA genes; *tRNA-Thr* for SfI and *tRNA-Pro* for SfIC (Figure 7). The insertion of lambdoid and P4-like phages usually occurs by site-specific recombination within the 3’-end of *tRNA* genes [48]. The 3’-end of *tRNA* gene possesses a conserved “CCA” codon which provides initial recognition for phage integrase and additionally, stable tRNA secondary structure and presence of multiple *tRNA* genes within a bacterial genome enhance phage integration [49]. The presence of a common SfI phage region in serotype 1c and serotype 1a/1b has raised an obvious question about the origin of serotype 1a and 1b by either deletion (loss of function) of *gtrIc* gene cluster from an ancestral serotype 1c strain or origin of serotype 1c from an ancestral serotype 1a and 1b by the acquisition of SfIC phage. The results presented here enabled us to answer this question by providing a complete overview of the origin of these serotypes using high-resolution whole-genome sequence analysis.

### 4.1. S. flexneri Serotype 1c Was Independently Evolved Twice from Serotype 1a and 1b Strains

The serotype switching due to deletion or disruption of the O-antigen modifying gene cluster is not uncommon in *S. flexneri.* For example, the *S. flexneri* serotype Y strain SFL124 originated from serotype 2a background due to the inactivation of the *gtrII* gene (from SfII phage) by an IS1 element [50]. However, our phylogenetic analysis showed that the Bangladesh-specific 1c strains (1c-α) and the remaining 1c strains (1c-β from the UK, Egypt, and Vietnam) were independently derived from the serotypes 1a and 1b, respectively. This is corroborated by the fact that all serotype 1c strains have remnants of SfI phage and hence intact *gtrI-*gene cluster but none of the serotype 1a and 1b strains had any of the genes from cryptic SfIC phage, suggesting that all the serotype 1c are derived from either serotype 1a strains or serotype 1b strains. However, not all the serotype 1a and 1b strains are clonal or derived from single ancestral strains. The clustering of serotype 1a and 1b strains into more than one sub-lineages suggests that these serotype 1a and 1b strains have evolved independently. Furthermore, some of the serotype 1a strains contain a complete SfI prophage sequence (SFL2494, UK and strain 0439, China) suggesting that these *S. flexneri* strains have acquired SfI phage relatively recently compared to the other serotype 1a and 1b strains or serotype 1c strains with cryptic SfI phage. The prophage genes that provide selective benefit to the bacterial host are often retained while the other prophage genes, which are not useful for the bacterial host get deleted over time resulting in a cryptic prophage [51]. Furthermore, DNA homology between phage residing on the same host can lead to homologous recombination resulting in host genome rearrangement which appears to be the case of Sf101 phage carrying *oacB* gene, which is present in serotype 1a, 1b and 1c strains [52].

The conventional classification of *S. flexneri* into different serotypes is based on group and type antisera specific to the O-antigenic variation. Hence, it is obvious to have multiple serotypes in one clade, which is based on the core set of genes. Interestingly, our phylogeny based on the core genes resulted in two populations of *S. flexneri* serotype 1c: 1c-α is specific to Bangladesh and 1c-β consists of strains from the UK, Egypt, and Vietnam. The previous study on serotype 1c, based on the Southern hybridization analysis had suggested that all these serotype 1c strains likely to have originated from a single ancestral 1a strain [53]. However, our study suggested that these strains have originated from at least two ancestral serotype 1a and 1b clones. These two ancestral *S. flexneri* 1a and 1b strains acquired SfIC bacteriophage that integrated into the *tRNA-Pro* gene resulting in serotype 1c-specific modification.

### 4.2. Changing Landscape in Drug Resistance of S. flexneri

We identified several antimicrobial-resistant genes in the majority of the sequenced strains. These isolates contained one or more genes that conferred resistance against sulphonamide, aminoglycosides, quinolone, trimethoprim, tetracycline, chloramphenicol, and beta-lactamase. These genotypes were consistent with the phenotypes tested by antibiotic susceptibility tests. Additionally, when examining the drug resistance of current drugs used in shigellosis treatment, most isolates were either resistant or intermediately resistant to one or more tested drugs. Interestingly, nalidixic acid (NA), which had previously been discontinued due to the prevalence of high resistance, had lower levels of resistance in the populations tested in this study than any of the currently deployed drugs. NA was used as a first-line drug for shigellosis treatment since November 1981 after high levels of drug resistance against ampicillin, chloramphenicol, tetracycline and trimethoprim/sulfamethoxazole was shown by *Shigella* strains [54]. However, by 1990, a large number of *Shigella* strains, *S. dysenteriae* type 1 in particular, acquired resistance to NA [55]. The clinical trials during 1988–1990 with ceftriaxone and ciprofloxacin in Bangladesh and Israel respectively, were found to be highly efficacious against severe shigellosis cases and were recommended as the drug of choice for shigellosis treatment [56,57]. In 2005, the WHO recommended the use of ciprofloxacin as the first-line and ceftriaxone, pivmecillinam and azithromycin as the second-line of drugs for the treatment of shigellosis [47]. However, in-line with our findings, the resistance of *Shigella* strains against WHO-recommended drugs such as ciprofloxacin and pivmecillinam has been growing since 2007 [58]. These results highlight the importance of drug resistance surveillance in the endemic region for the effective management of shigellosis.

## 5. Conclusions

Our study provided a clear understanding of the evolution of *S. flexneri* serotype 1c. *S. flexneri* serotype 1c possesses a highly complex O-antigen structure, modified by up to three different bacteriophages. The *S. flexneri* pangenome consists of a large group of homologous genes, only 40.6% of which are common in all the compared isolates. This suggests that these pathogens are capable of continued niche adaptation and can produce divergent populations. The phylogenetic analysis identified two major lineages of *S. flexneri* serotype 1c; one specific to Bangladesh and the other consisting of the isolates of the UK, Egypt, and Vietnam. The presence of multidrug resistance genes in the majority of isolates is alarming, and resistance to the majority of the current WHO-recommended drugs is a serious concern. The widespread distribution and the rise of multidrug resistance *S. flexneri* serotype 1c in most developing countries highlight the importance of a safe and efficacious *Shigella* vaccine, effective against common *S. flexneri* serotypes, including serotype 1c.

## Data Availability

The sequence read data generated in this study are available from GenBank Short Read Archive under the accession PRJNA606155.

## Supplementary Materials

Supplementary data are available online at https://www.mdpi.com/2073-4425/11/9/1042/s1, Figure S1: Integration of SfI phage. The end-trimmed reads from strains of serotype 1a, 1b, Y/Yv and 1c were mapped with the reference *S. flexneri* serotype 1a strain 0439, which has a complete copy of SfI phage. The image shows the coverage of the SfI phage region of the reference. Only representative samples are shown in the image due to window size limitation. Figure S2. SfIC phage integration. A. Cryptic SfIC phage region in Y394. The direction of arrows indicates the orientation of open reading frames (ORFs). The color code denotes the ORF types: red, hypothetical protein; plum, transposase and IS elements; green, tRNA and integrase; gray/black, annotated proteins and blue, *gtr* genes. B. Coverage of cryptic SfIC phage region in 1a, 1b, Y and 1c strains using Y394 as a reference. Only representative samples are shown in the image due to window size limitation. Figure S3. Pangenome accumulation curve of *S. flexneri*. The X-axis indicates the total number of genomes and Y-axis shows the number of genes - conserved vs total in *S. flexneri* pangenome. Figure S4. Maximum-likelihood (ML) phylogenetic tree with branch lengths drawn to scale. The ML tree was generated using the alignment of concatenated core genes with the recombination region removed. The general time-reversible model with a proportion of invariable sites (GTR+F+I) was used. The isolates from different sources were color-coded: red (Bangladesh), blue (Egypt), orange (Japan), green (UK), purple (Vietnam), and black (GenBank). The branch color represents the bootstrap support values of 1000 pseudoreplicates. The scale bar represents substitution per site. Table S1: List of bacterial strains used in the study. Table S2: List of *S. flexneri* core genes with reference to *S. flexneri* serotype 1c strain Y394.
