# Supplementary material for: Newly Emerged Serotype 1c of Shigella flexneri: Multiple Origins and Changing Drug Resistance Landscape
Source: Genes (Basel). 2020 Sep 3;11(9):1042. doi: 10.3390/genes11091042 (PMC7565858; doi:10.3390/genes11091042)
Supplement: Supplementary file 1 [file genes-11-01042-s001.zip › Supplimentary Materials_file 1.docx]

**Supplementary Materials**

**Supplementary Figure S1: Integration of SfI phage.** The end-trimmed reads from strains of serotype 1a, 1b, Y/Yv and 1c were mapped with the reference *S. flexneri* serotype 1a strain 0439, which has a complete copy of SfI phage. The image shows the coverage of the SfI phage region of the reference. Only representative samples are shown in the image due to window size limitation.

**Supplementary Figure S2. SfIC phage integration.** A. Cryptic SfIC phage region in Y394. The direction of arrows indicates the orientation of open reading frames (ORFs). The color code denotes the ORF types: red, hypothetical protein; plum, transposase and IS elements; green, tRNA and integrase; gray/black, annotated proteins and blue, *gtr* genes. B. Coverage of cryptic SfIC phage region in 1a, 1b, Y and 1c strains using Y394 as a reference. Only representative samples are shown in the image due to window size limitation.

**Supplementary Figure S3. Pangenome accumulation curve of *S. flexneri*.** The X-axis indicates the total number of genomes and the Y-axis shows the number of genes - conserved vs total in *S. flexneri* pangenome.

**Supplementary Figure S4. Maximum-likelihood (ML) phylogenetic tree with branch lengths drawn to scale.** The ML tree was generated using the alignment of concatenated core genes with the recombination region removed. The general time-reversible model with a proportion of invariable sites (GTR+F+I) was used. The isolates from different sources were color-coded: red (Bangladesh), blue (Egypt), orange (Japan), green (UK), purple (Vietnam) and black (GenBank). The branch color represents the bootstrap support values of 1,000 pseudoreplicates. The scale bar represents substitution per site.

**Supplementary Table S1: List of bacterial strains used in the study.**

| **Supplementary Figure S1**  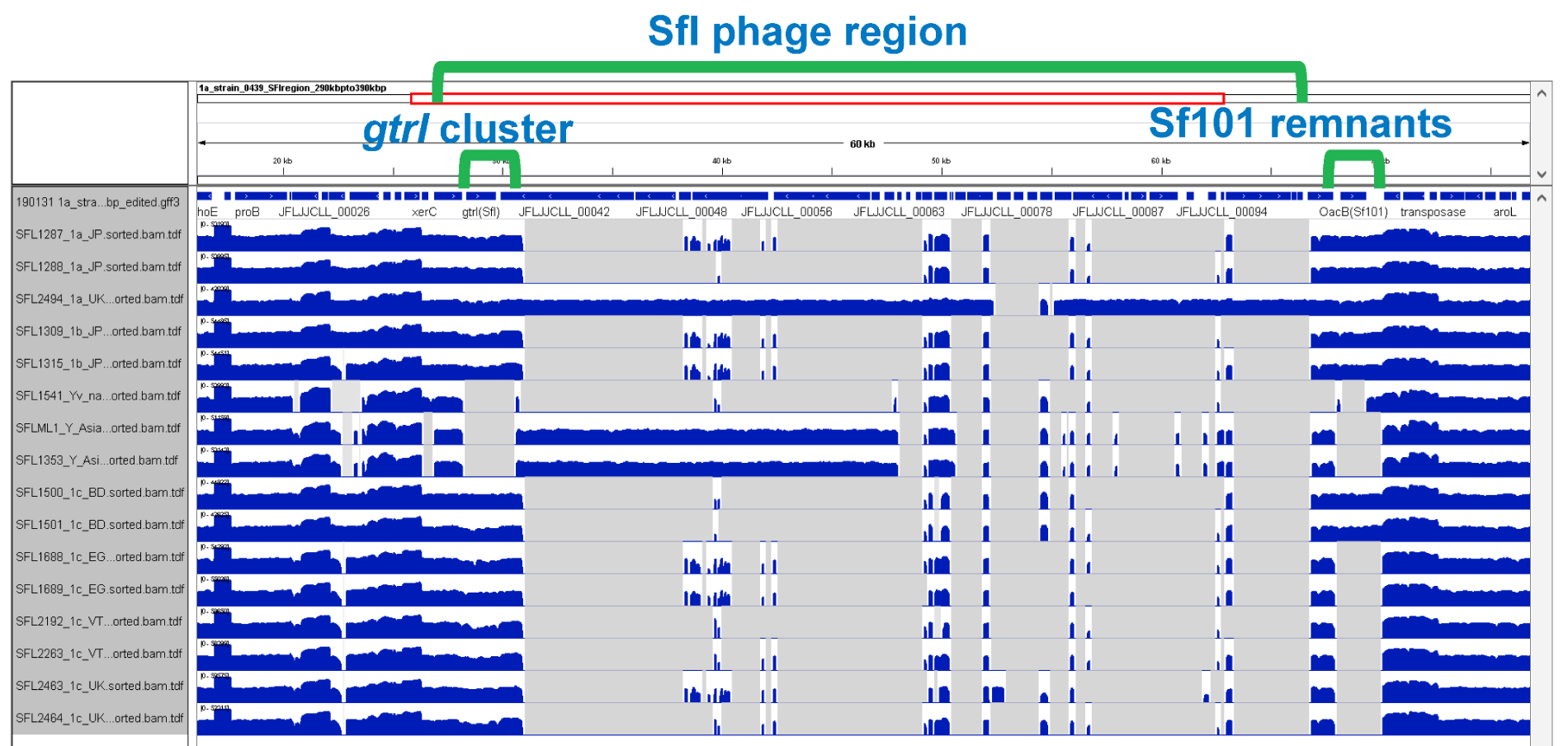  **Supplementary Figure S2**  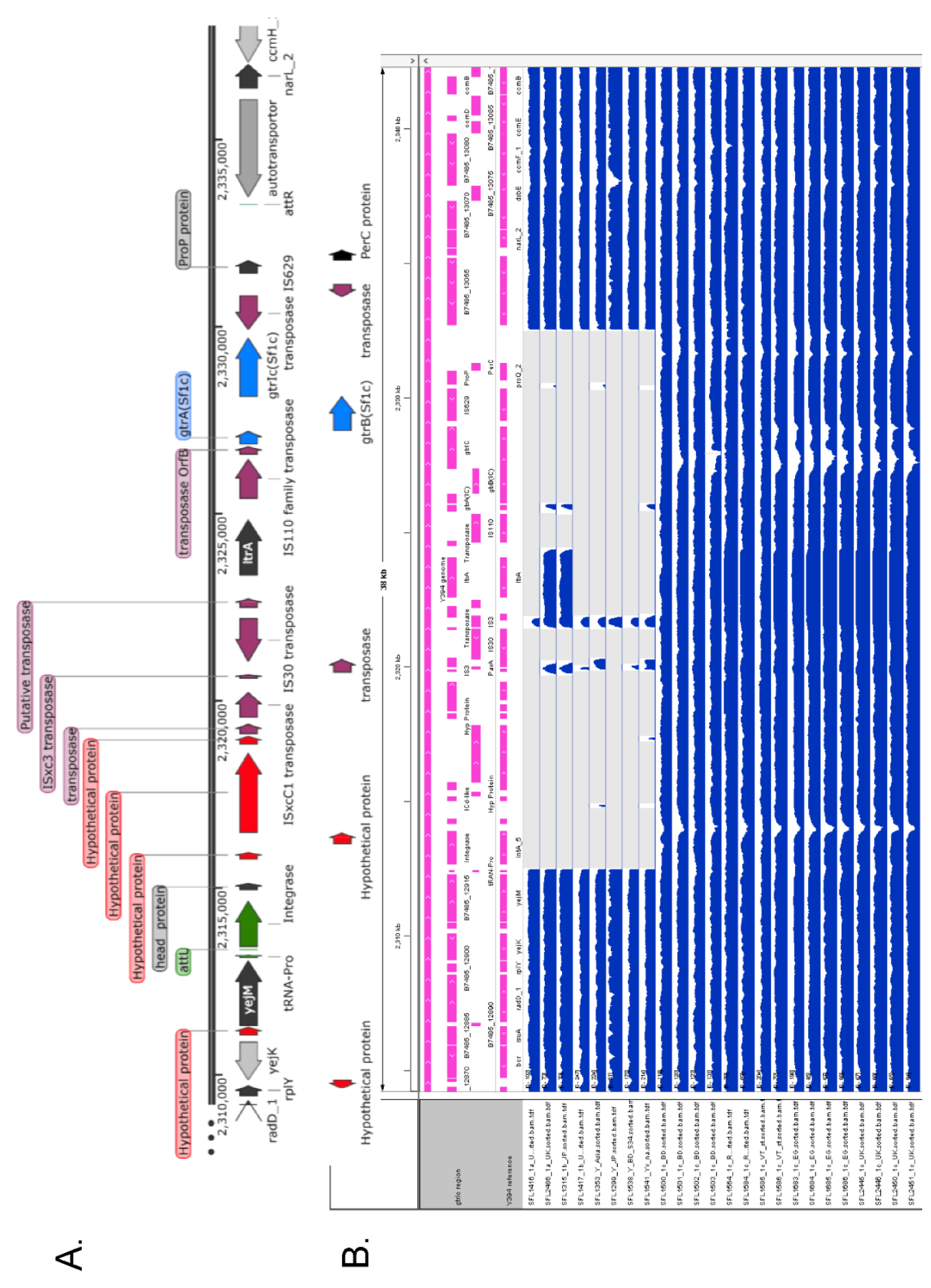 |  |
| --- | --- |
| **Supplementary Figure S3**  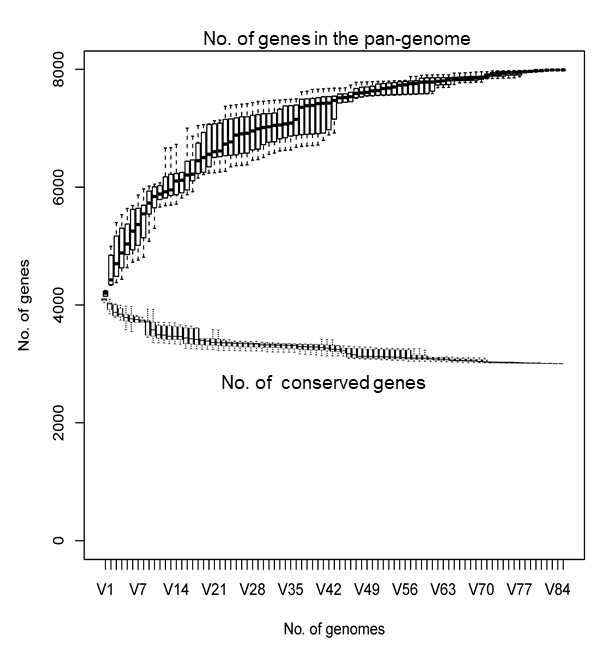 |  |

**Supplementary Figure S4.**


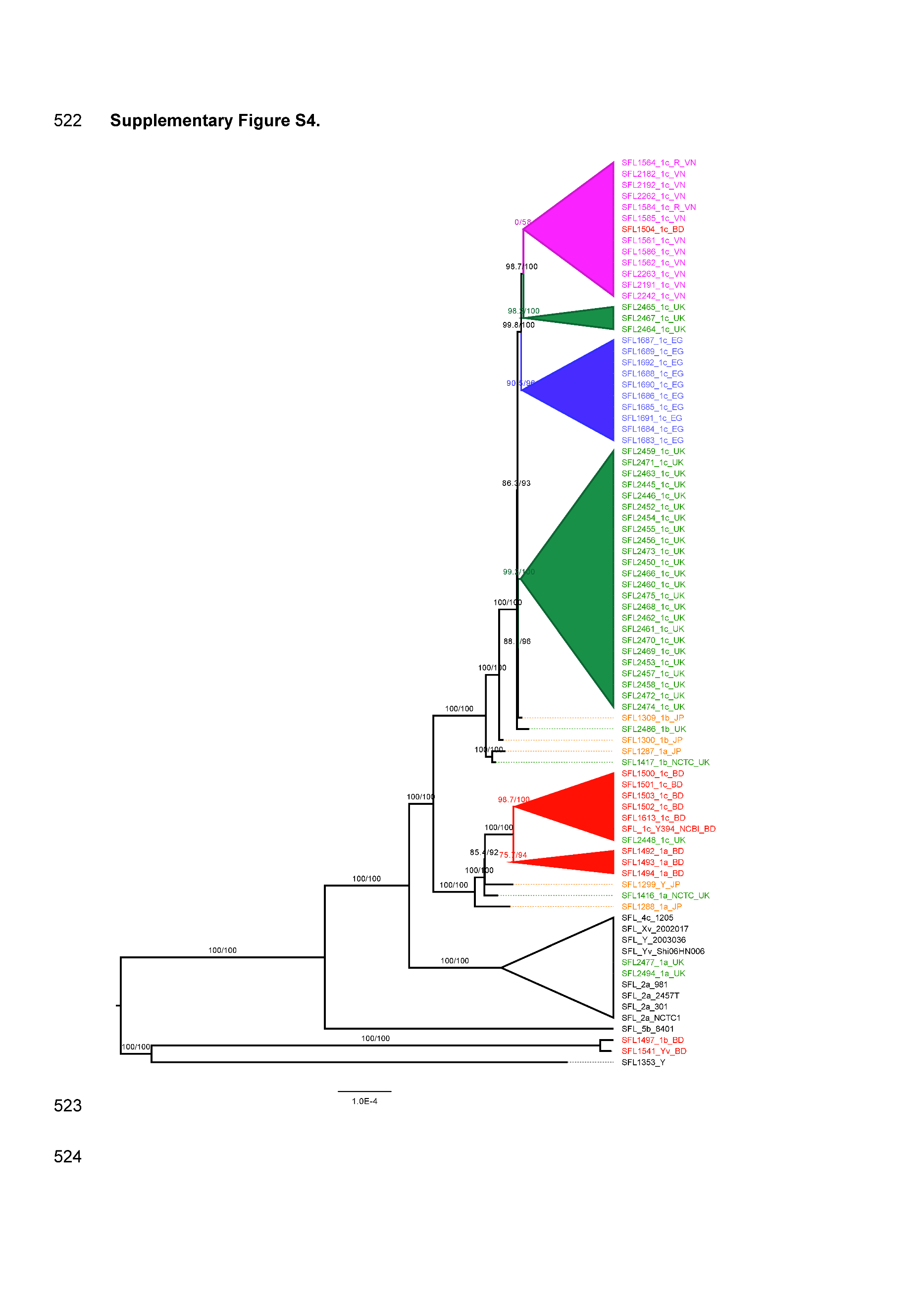


**Supplementary Table S1:** **List of bacterial strains used in the study.**

| **Strain** | **Serotype** | **Country of Isolation** | **Source/Reference^Ψ^** |
| --- | --- | --- | --- |
| SFL1287 | 1a | Japan | Sasakawa |
| SFL1288 | 1a | Japan | Sasakawa |
| SFL1299 | Y | Japan | Sasakawa |
| SFL1300 | 1b | Japan | Sasakawa |
| SFL1309 | 1b | Japan | Sasakawa |
| SFL1315 | 1b | Japan | Sasakawa |
| SFL1353/SFL124 | Y | Srilanka  (Tourist from Sweden) | (Lindberg et al. 1988) |
| SFL1416 | 1a | NCTC, UK | NCTC, UK |
| SFL1417 | 1b | Japan | NCTC, UK |
| SFL1492 | 1a | Bangladesh | NCTC, UK |
| SFL1493 | 1a | Bangladesh | ICDDRB |
| SFL1494 | 1a | Bangladesh | ICDDRB |
| SFL1496 | 1b | Bangladesh | NCTC, UK |
| SFL1497 | 1b | Bangladesh | ICDDRB |
| SFL1500 | 1c | Bangladesh | ICDDRB |
| SFL1501 | 1c | Bangladesh | ICDDRB |
| SFL1502 | 1c | Bangladesh | ICDDRB |
| SFL1503 | 1c | Bangladesh | ICDDRB |
| SFL1504 | 1c | Bangladesh | ICDDRB |
| SFL1538 | Y | Bangladesh | ICDDRB |
| SFL1541 | Yv | Bangladesh | ICDDRB |
| SFL1561 | 1c | Vietnam | P. D. Cam |
| SFL1562 | 1c | Vietnam | P. D. Cam |
| SFL1564 | 1c(Rough) | Vietnam | P. D. Cam |
| SFL1565 | 1c | Vietnam | P. D. Cam |
| SFL1566 | 1c | Vietnam | P. D. Cam |
| SFL1567 | 1c | Vietnam | P. D. Cam |
| SFL1572 | 1c | Vietnam | P. D. Cam |
| SFL1573 | 1c | Vietnam | P. D. Cam |
| SFL1578 | 1c | Vietnam | P. D. Cam |
| SFL1579 | 1c | Vietnam | P. D. Cam |
| SFL1580 | 1c | Vietnam | P. D. Cam |
| SFL1581 | 1c | Vietnam | P. D. Cam |
| SFL1582 | 1c | Vietnam | P. D. Cam |
| SFL1584 | 1c(Rough) | Vietnam | P. D. Cam |
| SFL1585 | 1c | Vietnam | P. D. Cam |
| SFL1585 | 1c | Vietnam | P. D. Cam |
| SFL1613 (Y394) | 1c | Bangladesh | ICDDRB |
| SFL1683 | 1c | Egypt | (Atef El-Gendy et al. 1999b) |
| SFL1684 | 1c | Egypt | (Atef El-Gendy et al. 1999b) |
| SFL1685 | 1c | Egypt | (Atef El-Gendy et al. 1999b) |
| SFL1685 | 1c | Egypt | (Atef El-Gendy et al. 1999b) |
| SFL1687 | 1c | Egypt | (Atef El-Gendy et al. 1999b) |
| SFL1688 | 1c | Egypt | (Atef El-Gendy et al. 1999b) |
| SFL1689 | 1c | Egypt | (Atef El-Gendy et al. 1999b) |
| SFL1690 | 1c | Egypt | (Atef El-Gendy et al. 1999b) |
| SFL1691 | 1c | Egypt | (Atef El-Gendy et al. 1999b) |
| SFL1692 | 1c | Egypt | (Atef El-Gendy et al. 1999b) |
| SFL2182 | 1c | Vietnam | P. D. Cam |
| SFL2191 | 1c | Vietnam | P. D. Cam |
| SFL2192 | 1c | Vietnam | P. D. Cam |
| SFL2242 | 1c | Vietnam | P. D. Cam |
| SFL2262 | 1c | Vietnam | P. D. Cam |
| SFL2263 | 1c | Vietnam | P. D. Cam |
| SFL2445 | 1c | United Kingdom | PHE |
| SFL2446 | 1c | United Kingdom | PHE |
| SFL2447 | 1c | United Kingdom | PHE |
| SFL2448 | 1c | United Kingdom | PHE |
| SFL2449 | 1c | United Kingdom | PHE |
| SFL2450 | 1c | United Kingdom | PHE |
| SFL2451 | 1c | United Kingdom | PHE |
| SFL2452 | 1c | United Kingdom | PHE |
| SFL2453 | 1c | United Kingdom | PHE |
| SFL2454 | 1c | United Kingdom | PHE |
| SFL2455 | 1c | United Kingdom | PHE |
| SFL2456 | 1c | United Kingdom | PHE |
| SFL2457 | 1c | United Kingdom | PHE |
| SFL2458 | 1c | United Kingdom | PHE |
| SFL2459 | 1c | United Kingdom | PHE |
| SFL2460 | 1c | United Kingdom | PHE |
| SFL2461 | 1c | United Kingdom | PHE |
| SFL2462 | 1c | United Kingdom | PHE |
| SFL2463 | 1c | United Kingdom | PHE |
| SFL2464 | 1c | United Kingdom | PHE |
| SFL2465 | 1c | United Kingdom | PHE |
| SFL2466 | 1c | United Kingdom | PHE |
| SFL2467 | 1c | United Kingdom | PHE |
| SFL2468 | 1c | United Kingdom | PHE |
| SFL2469 | 1c | United Kingdom | PHE |
| SFL2470 | 1c | United Kingdom | PHE |
| SFL2471 | 1c | United Kingdom | PHE |
| SFL2472 | 1c | United Kingdom | PHE |
| SFL2473 | 1c | United Kingdom | PHE |
| SFL2474 | 1c | United Kingdom | PHE |
| SFL2475 | 1c | United Kingdom | PHE |
| SFL2477 | 1a | United Kingdom | PHE |
| SFL2485 | 1b | United Kingdom | PHE |
| SFL2494 | 1a | United Kingdom | PHE |

Ψ

Sasakawa- Prof. C. Sasakawa, Department of Microbiology and Immunology, Institute of Medical Science, University of Tokyo, Japan.

P.D. Cam- Professor, Department of Microbiology, National Institute of Hygiene and Epidemiology, Hanoi, Vietnam

NCTC- The National Collection of Type Cultures, London, UK

ICDDRB- International Centre for Diarrhoeal Disease Research, Bangladesh

PHE- Public Health England (Gastrointestinal Bacterial Reference Unit, National Infection Service, PHE, London, NW9 5HT, United Kingdom)
